# Immune regulation and lymphangiogenesis by lymphatic endothelial cells in the decidua in severe preeclampsia

**DOI:** 10.1038/s41598-026-35667-3

**Published:** 2026-01-13

**Authors:** Suhra Kim, Yeji Lee, Ja-Young Kwon, Yong-Sun Maeng

**Affiliations:** Department of Obstetrics and Gynecology, Institute of Women’s Life Medical Science, Yonsei University College of Medicine, 250 Seongsanno, Seodaemun-gu, Seoul, 03722 Korea

**Keywords:** Placenta, Lymphangiogenesis, Decidual lymphatic endothelial cells, Preeclampsia, Pregnancy, Cell biology, Diseases, Immunology, Medical research

## Abstract

Lymphatic vasculature regulates lymphocyte trafficking and modulates adaptive immunity. Imbalanced immune cells at the maternal-fetal interface may contribute to severe preeclampsia (PE). Impaired placental lymphangiogenesis and immune dysregulation could contribute to PE but supporting evidence is limited. Here, we investigate the association between lymphangiogenesis and immune regulation in severe PE. First, we identified the presence of LYVE1-positive lymphatic vessels in the decidua, and then decidual lymphatic endothelial cells (dLECs) were isolated and cultured from chorioamniotic membranes obtained at cesarean section from women with PE (*n* = 15) and gestational age-matched controls (*n* = 15). The cells were identified by LYVE1, Prox1, and CD31 expression. Gene expression analysis showed the significant different gene expression profiles in PE compared to normal (lymphatic vessel development, immune cell trafficking and T-cell activation regulation). dLECs from PE pregnancies showed substantially reduced migration, adhesion, morphological differentiation, and decreased lymphatic sprouting in a 3D lymphatic ring assay compared with normal. Additionally, they exhibited low chemokine ligand 21 expression, impaired dendritic cell recruitment, and reduced Akt-eNOS-nitric oxide signaling, which suppresses decidual cytotoxic T-cell activation in decidua. Collectively, our findings suggest that impaired lymphatic vessel function and molecular alterations in the decidua may disrupt immune regulation and contribute to severe PE.

**Supplementary Information:**

The online version contains supplementary material available at 10.1038/s41598-026-35667-3.

## Introduction

Preeclampsia (PE), marked by hypertension and proteinuria after 20 weeks of gestation, affects 2–8% of pregnancies and remains a major cause of maternal and fetal morbidity and mortality worldwide^1,2^. PE is widely attributed to endothelial dysfunction, dysregulated angiogenesis, and inadequate trophoblast invasion, resulting in impaired spiral artery remodeling and poor placentation^3–8^. Increasing evidence implicates the maternal immune system, with excessive activation of both innate and adaptive immunity contributing to reduced immune tolerance^9–15^.

Immune tolerance during pregnancy is crucial, particularly at the fetal-maternal interface within the decidua. Proper immune regulation in the decidua is essential for pregnancy maintenance and is primarily mediated by the activation of regulatory T (Treg) cells, which suppress the activity of CD4 and CD8 T cells. This results in successful pregnancy maintenance. However, a reduction in Treg cells accompanied by an increase in CD4 and CD8 T cell activity disrupts immune regulation, leading to pregnancy complications and triggering various disorders^11,12,16–21^.

Recently, lymphatic vessels in the decidua have been recognized as a key factor for immune regulation during pregnancy, contributing to immune cell drainage and antigen presentation^16,22,23^. Although an earlier study suggested that lymphatic vessels disappear during decidualization^24^, this view has been challenged by reports demonstrating the presence lymphatic structures within the human decidua throughout gestation^16,25^. The potential involvement of these decidual lymphatic vessels in the pathogenesis of preeclampsia has also been proposed^26,27^. In our previous study, we confirmed these findings and demonstrated a significant reduction of decidual lymphatic vessels in patients with preeclampsia compared with controls^16^.

Lymphatic endothelial cells (LECs), which form lymphatic vessels in tissues and lymph nodes, interact with both innate and adaptive immune cells within these environments. LECs may help modulate inflammation and adaptive immune responses by regulating antigen presentation, immune cell trafficking, and lymphangiogenesis^28–30^. A recent study reported that LEC dysfunction leads to several pathological conditions. For instance, CD36 deletion in LECs may be associated with obesity and insulin resistance^31^. LECs exhibit a distinct metabolic profile, which in pathological lymphangiogenesis leads to lymphoedema and corneal graft rejection^32^. Additionally, autophagy in LECs regulates T cell activation, and its impairment exacerbates arthritis by promoting Th17 cell migration^33^.

Based on these findings, we hypothesized that dysfunctional LECs at the maternal-fetal interface impair immune regulation and lymphatic vessel development. Proinflammatory changes and abnormal lymphangiogenesis are closely associated with the pathogenesis of PE^16,34–36^.

In this study, we aimed to investigate the functional and genetic differences in lymphatic vessels between patients with PE and normal pregnancies and to explore the potential correlation between lymphatic vessel impairment and the development of PE.

Our findings provide evidence for the critical role of immune tolerance during pregnancy and highlight the importance of decidual lymphatic vessels in immune regulation. Understanding these functional and genetic alterations in preeclamptic mothers may offer new insights into the pathogenesis of PE and other pregnancy-related disorders, potentially paving the way for targeted therapies and interventions to promote healthy pregnancy outcomes.

## Results

### Isolation of decidual LECs (dLECs) from human decidua

**Fig. 1:**
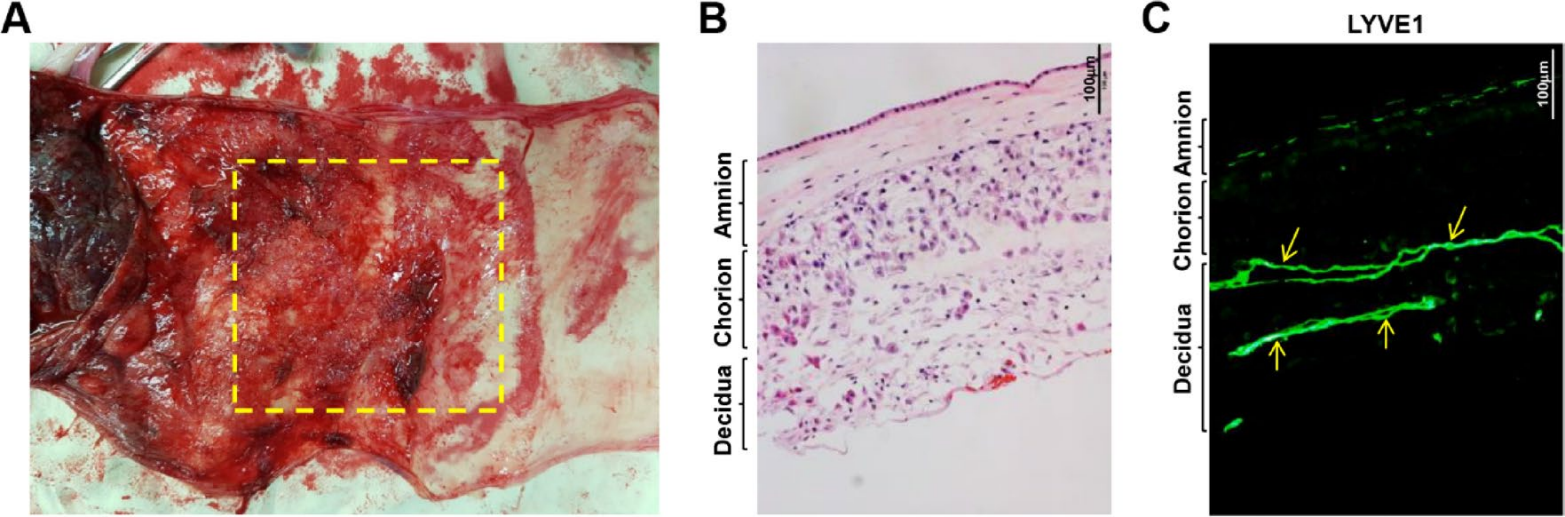
Identification of decidua lymphatic endothelial cells (dLECs) from human decidua. (**A**) Image of chorioamniotic membranes of human placenta. The yellow square indicates the decidual area between the placenta and amnion, where decidual tissue is most abundant. (**B**) Cross-section of chorioamniotic membranes with hematoxylin and eosin staining (Olympus CX33 microscope). Analysis of imaging data was conducted using Fiji/ImageJ software. Scale bar = 100 μm. (**C**) Human chorioamniotic membranes with abundant decidua stained for lymphatic vessels using anti-LYVE1 antibody and labeled with a FITC-conjugated secondary antibody. Arrows indicate LYVE1-positive lymphatic vessels in the human decidua. Images were viewed by using an Olympus IX81-ZDC microscope with a LUCPL FLN 10×/1.0 or 2.0 NA lens. Analysis of imaging data was conducted using Fiji/ImageJ software. Scale bar = 100 μm. LYVE1, lymphatic vessel endothelial hyaluronan receptor 1.

To compare the gene expression levels in dLECs derived from preeclamptic and normal pregnancies, we first analyzed the presence of lymphatic vessels at the maternal-fetal interface. Hematoxylin and eosin (H&E) staining of human samples revealed that the fetal chorioamniotic membranes and maternal decidua are closely located, forming a distinct interface (Fig. 1A,B). Immunofluorescence staining for lymphatic vessel endothelial hyaluronan receptor 1 (LYVE1), a lymphatic marker, showed no LYVE1-expressing structures in the amnion or chorion. However, LYVE1-positive vessels were observed exclusively in the decidua layer (Fig. 1C). These results show that lymphatic vessels are present only in the maternal decidua at the human maternal-fetal interface.

**Fig. 2:**
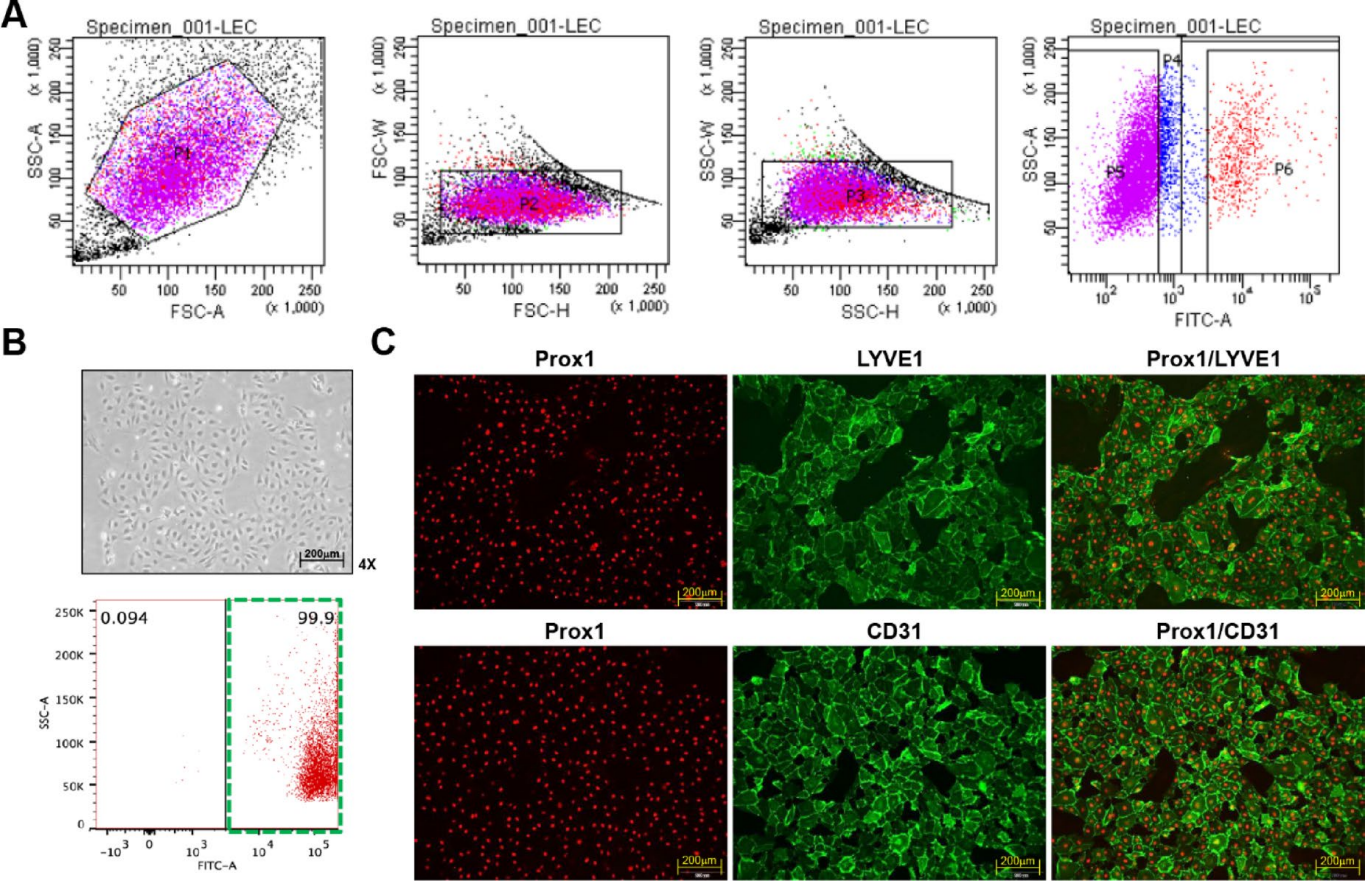
Isolation of dLECs from human decidua. (**A**) Flow cytometry analysis of the isolated cell from human decidua. Cells were labeled with FITC-conjugated LYVE1 antibody, and the forward scatter (FSC) against side scatter (SSC) was analyzed sequentially. Cells with LYVE1 expression were gated in the p6 region and cultured. (**B**) To evaluate the purity of cultured FACS-isolated LYVE1-positive cells, cells were labeled with FITC-conjugated LYVE1 antibody, and FACS analysis was performed. The green square of the dot plot showed the 99.9% LEC purity of the isolated cell from the decidua. (**C**) Immunofluorescence staining demonstrating the expression of LEC-specific markers PROX1 and LYVE1, as well as the endothelial-specific marker CD31. dLECs, decidual lymphatic endothelial cells; LYVE1, lymphatic vessel endothelial hyaluronan receptor 1; PROX1, Prospero homeobox protein 1.

After delivery, dLECs were isolated from decidual tissues using physical and enzymatic methods^16^ and purified by flow cytometry. Flow cytometry analysis confirmed that 99.9% of the isolated cells were dLECs (Fig. 2A,B). Immunofluorescence staining further validated dLEC identity, showing co-expression of lymphatic markers Prospero homeobox protein 1 (Prox1) and LYVE1, along with the endothelial marker CD31 (Fig. 2C). These results confirm the successful isolation of a highly pure dLEC population from the maternal-fetal interface.

### Origin of dLECs from human decidua

**Fig. 3:**
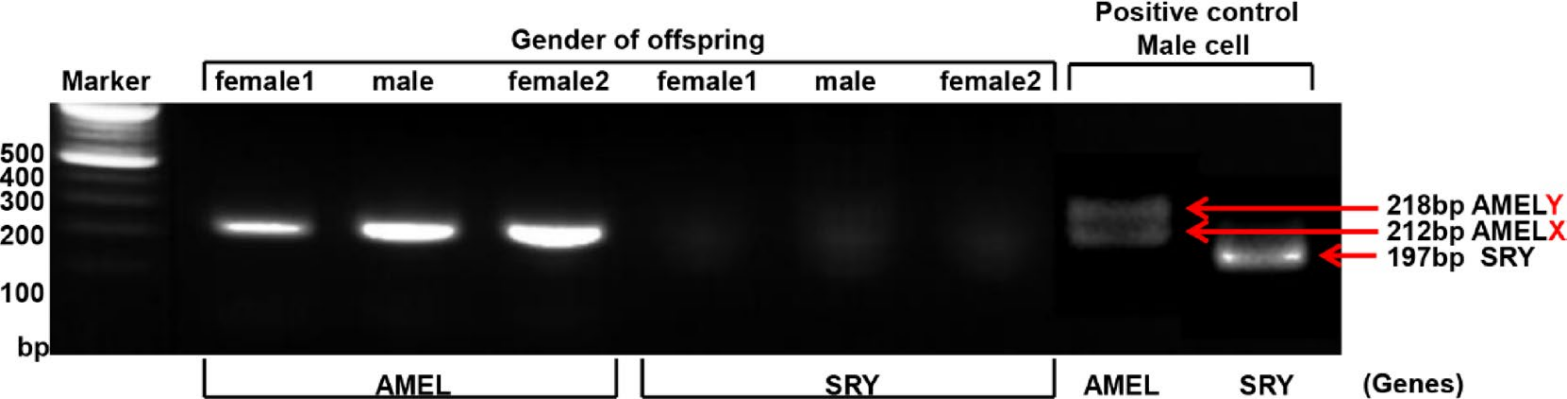
Confirmation of the maternal origin of isolated dLECs using qPCR. PCR amplification of the *AMEL* and *SRY* genes was performed on genomic DNA extracted from isolated dLECs. Human skin lymphatic endothelial cells from a male donor were used as a positive control. The control sample showed two *AMEL* bands (218 bp for *AMELX* and 212 bp for *AMELY*) and a single *SRY* band at 197 bp. In contrast, all three dLEC samples (two from pregnancies with female fetuses and one with a male fetus) exhibited only the *AMELX* band (212 bp) without *SRY* amplification, confirming that the isolated cells were of maternal origin. dLECs, decidual lymphatic endothelial cells; AMEL, Amelogenin; SRY, sex-determining region Y.

To confirm that the isolated dLECs from the decidua were of maternal origin, we performed polymerase chain reaction (PCR)-based amplification of the Amelogenin *(AMEL)* and sex-determining region Y (*SRY*) genes using genomic DNA (gDNA). Sex-specific human skin LECs (male) were used as a positive control. In the positive control, amplification of the *AMEL* gene produced two bands (218 and 212 bp), and *SRY* gene amplification yielded a single band of 197 bp in males (Fig. 3). We analyzed three dLEC samples: two from pregnancies with female offspring and one from a male offspring. All three dLEC samples showed a single *AMEL* band at 212 bp and no detectable *SRY* band (Fig. 3), indicating a female genotype. These results confirm that the isolated dLECs were of maternal origin in all cases.

### Gene expression comparison analysis of normal dLECs (N-dLECs) and pre-eclamptic dLECs (PE-dLECs)

**Fig. 4:**
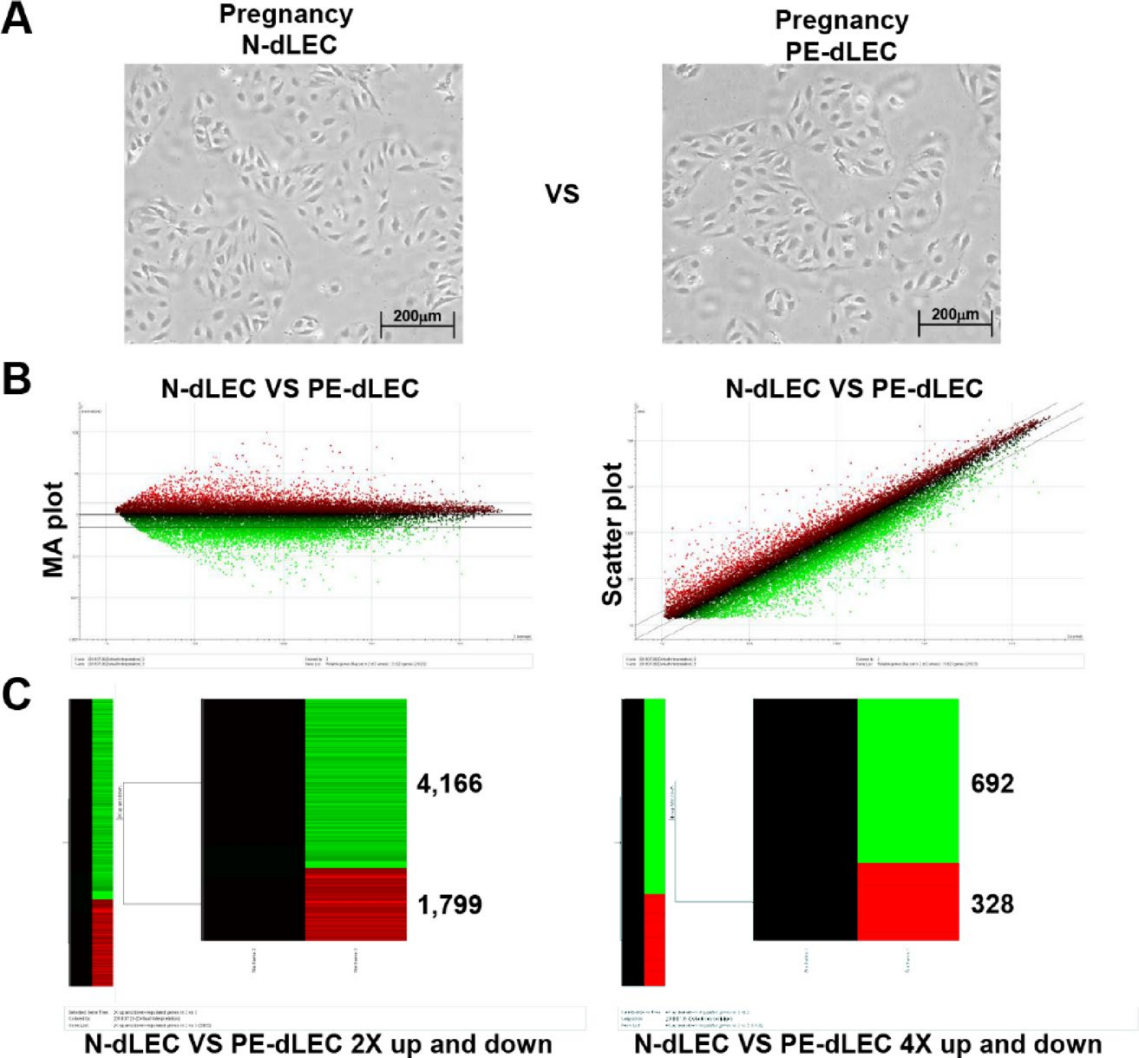
Gene expression profiling of dLECs from normal (N-dLECs) and preeclamptic (PE-dLECs) pregnancies. (**A**) Cultivation of N-dLECs and PE-dLECs. (**B**) QuantSeq3’mRNA sequencing analysis was performed to compare gene expression profiles of dLECs isolated from preeclamptic pregnancies (PE-dLECs, *n* = 3) and normal pregnancies (N-dLECs, *n* = 3). MA plot (M-value: Log-ratio, A-value: Average intensity) and scatter plot analysis of microarray data. (**C**) Hierarchical cluster analysis of differentially expressed (twofold or fourfold) genes in PE-dLECs and N-dLECs. A total of 4,166 genes were significantly upregulated and 1,799 genes were downregulated (fold change > 2.0, *p* < 0.05) in PE-dLECs compared with N-dLECs. Among these, 692 genes showed more than a fourfold increase, and 328 genes were downregulated by more than fourfold (fold change > 4.0, *p* < 0.05). Data represent mean values from three biological replicates per group, analyzed using standard RNA-seq normalization and differential expression pipelines. These results indicate widespread alterations in gene expression in dLECs associated with preeclampsia. dLECs, decidual lymphatic endothelial cells; N-dLECs, dLECs from normal pregnancies; PE-dLECs, dLECs from preeclamptic pregnancies.

We performed QuantSeq 3′ mRNA sequencing to compare gene expression profiles in N-dLECs and PE-dLECs. The analysis revealed that 4,166 genes were upregulated and 1,799 genes downregulated by more than twofold (fold change > 2.0, *p* < 0.05). Additionally, 692 genes were upregulated and 328 genes were downregulated by more than fourfold, respectively (fold change > 4.0, *p* < 0.05) (Fig. 4A–C). These findings indicate widespread gene expression changes in dLECs from preeclamptic pregnancies compared with those from normal pregnancies.

**Fig. 5:**
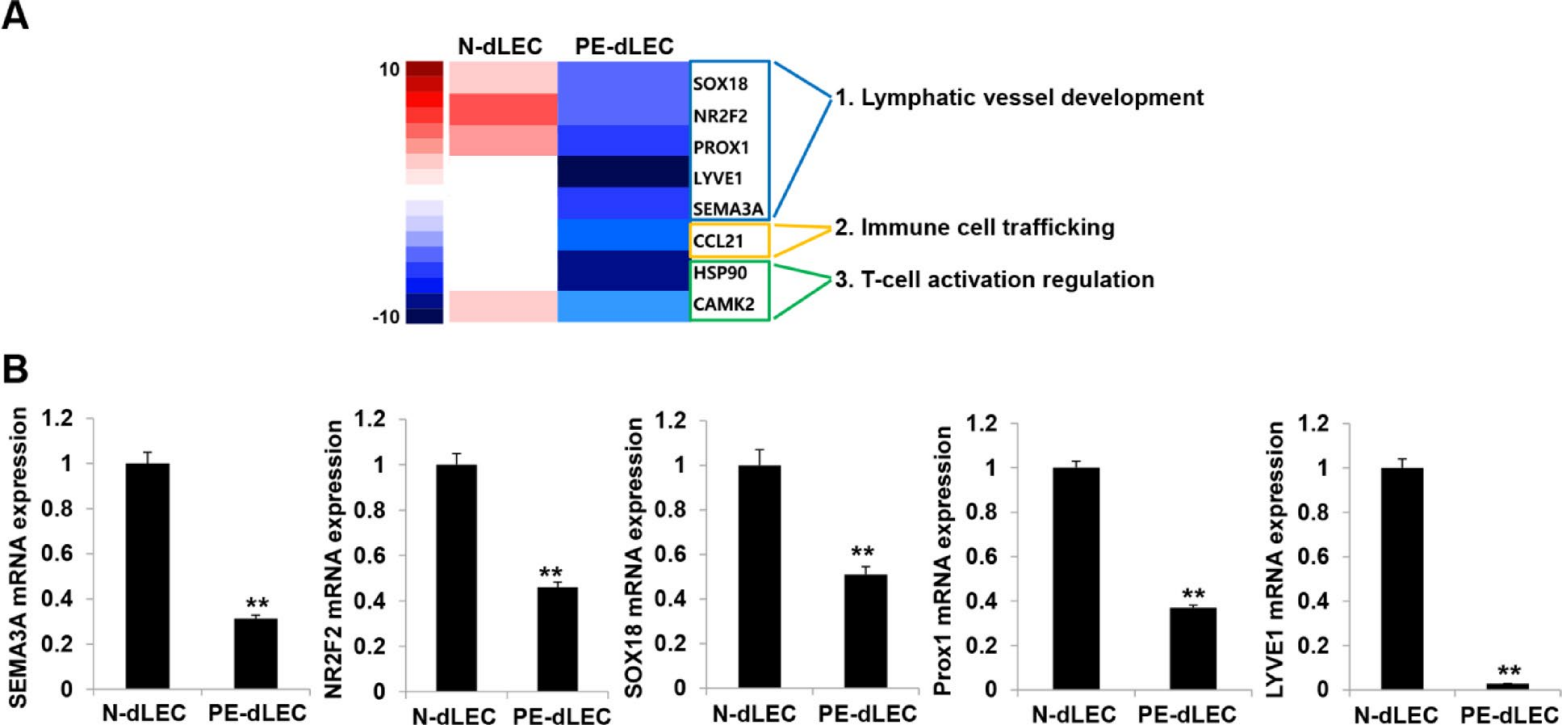
Gene expression analysis of dLECs from normal (N-dLECs) and preeclamptic (PE-dLECs) pregnancies. (**A**) Heatmap showing a cluster of genes that were downregulated more than twofold in PE-dLECs (*n* = 3) compared with N-dLECs (*n* = 3) based on RNA-sequencing analysis. (**B**) RT-qPCR analysis showed that lymphatic vessel development-related genes were significant decrease in PE-dLECs compared with N-dLECs. Each experiment was performed in biological triplicates, and data are presented as mean ± SEM. Statistical significance was assessed using one-way ANOVA followed by Tukey’s test. **, *p* < 0.01 vs. N-dLEC. dLECs, decidual lymphatic endothelial cells; N-dLECs, dLECs from normal pregnancies; PE-dLECs, dLECs from preeclamptic pregnancies; RT-qPCR, quantitative reverse-transcription PCR; SEM, standard error of the mean.

Among the significantly dysregulated genes, many of the downregulated genes were associated with lymphatic vessel development, immune cell trafficking, and T-cell activation (Fig. 5A). Genes such as *SOX18*, nuclear receptor 2F2 (*NR2F2*), *PROX1*, *LYVE1*, semaphorin-3 A (*SEMA3A*, lymphatic vessel development), chemokine (C-C motif) ligand 21 (*CCL21*, immune cell trafficking), and heat shock protein 90 (*HSP90*) and Ca²⁺/Calmodulin-Dependent Protein Kinase II (*CAMKII*, T-cell activation) were significantly downregulated in dLECs from preeclamptic pregnancies. These results were further validated by quantitative reverse-transcription PCR (RT-qPCR), which confirmed reduced expression of these genes in PE-dLECs (Fig. 5B). Collectively, these results suggest that lymphangiogenic activity and immune regulatory functions may be suppressed in PE-dLECs compared with N-dLECs.

### Decreased lymphangiogenic function of PE-dLECs

**Fig. 6:**
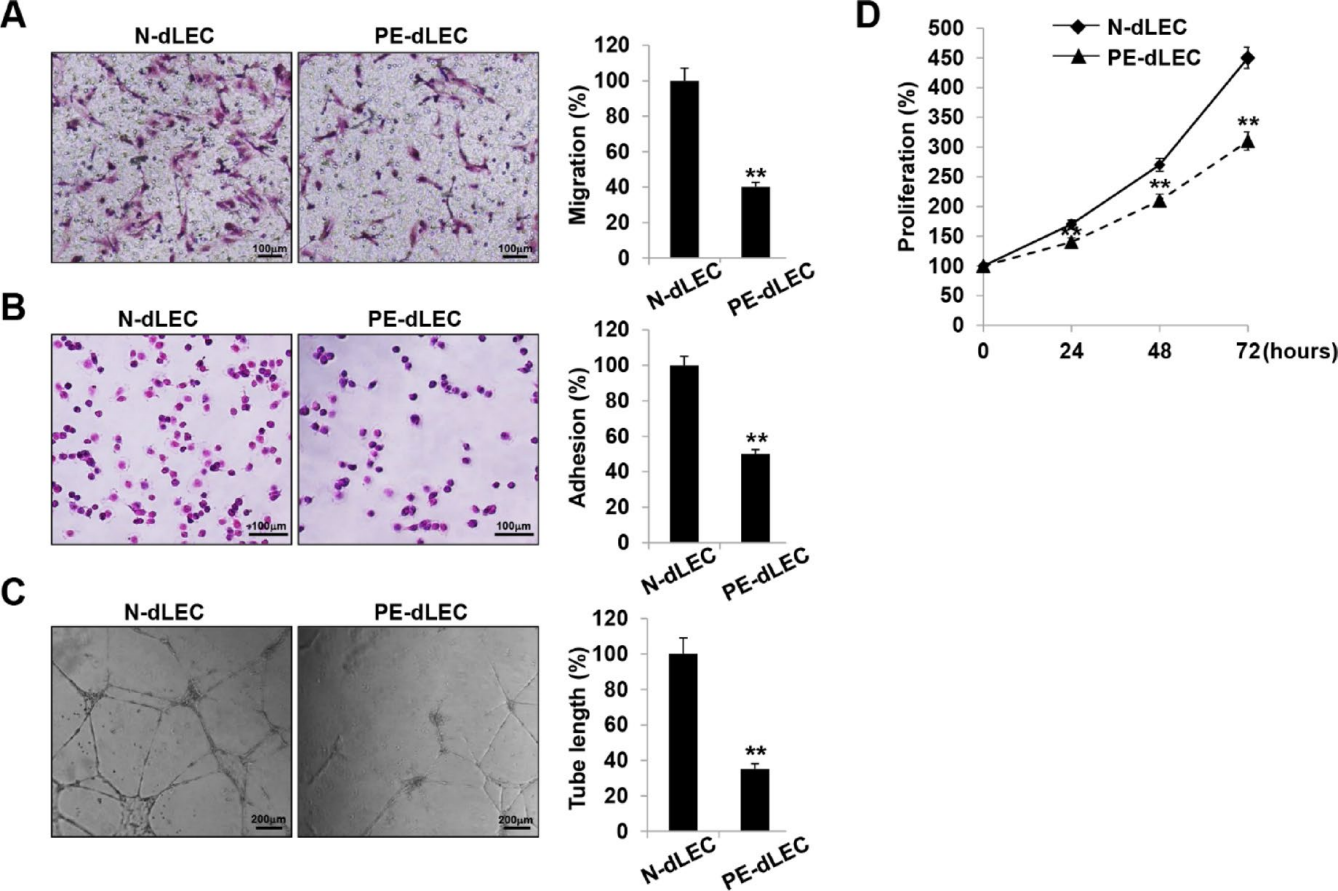
Decreased lymphangiogenic activity in PE-dLECs. (**A**) Cell migration was quantified by counting the number of cells that migrated to the lower surface of a Transwell membrane, observed under optical microscopy at 200× magnification. Migration of PE-dLECs was markedly reduced compared with N-dLECs. (**B**) For adhesion analysis, a 96-well plate was coated overnight at 4 °C with 0.1 mg/mL human fibronectin. dLECs in an adhesion buffer were seeded at 10^5^ cells/well in a 100-µL volume and incubated for 30 min at 37 °C. Cell adhesion was quantified by counting the cells that attached to the fibronectin-coated matrix using optical microscopy at 200× magnification. Fewer PE-dLECs adhered to the fibronectin-coated surface compared with N-dLECs. (**C**) Tubular network formation of the dLECs from normal and preeclamptic pregnancies was assessed on Matrigel and imaged at 24 h. PE-dLECs showed impaired tube formation with fewer and shorter tubular structures. (**D**) Cell proliferation was evaluated using an MTT assay. Absorbance value (OD) of each well was measured at 490 nm at 24 h, 48 h, and 72 h, demonstrating a significantly lower proliferation rate in PE-dLEC than in N-dLEC. All experiments were performed in triplicate with three different cell lines. Data are presented as mean ± SEM, and Statistical significance was assessed using one-way ANOVA followed by Tukey’s test. **, *p* < 0.01 vs. N-dLEC. N-dLECs, dLECs from normal pregnancies; PE-dLECs, dLECs from preeclamptic pregnancies; dLECs, decidual lymphatic endothelial cell; MTT, 3-(4,5-dimethylthiazol-2-yl)-2,5-diphenyl tetrazolium bromide; OD, optical density.

We investigated the in vitro lymphangiogenic function of dLECs to ascertain whether the reduced expression of lymphangiogenic genes in PE leads to functional impairment. Compared with dLECs from the normal pregnancy group, dLECs from preeclamptic pregnancies showed reduced migration, adhesion, and tube formation (Fig. 6A–C). Additionally, their proliferation was lower at 24, 48, and 72 h (Fig. 6D).

**Fig. 7:**
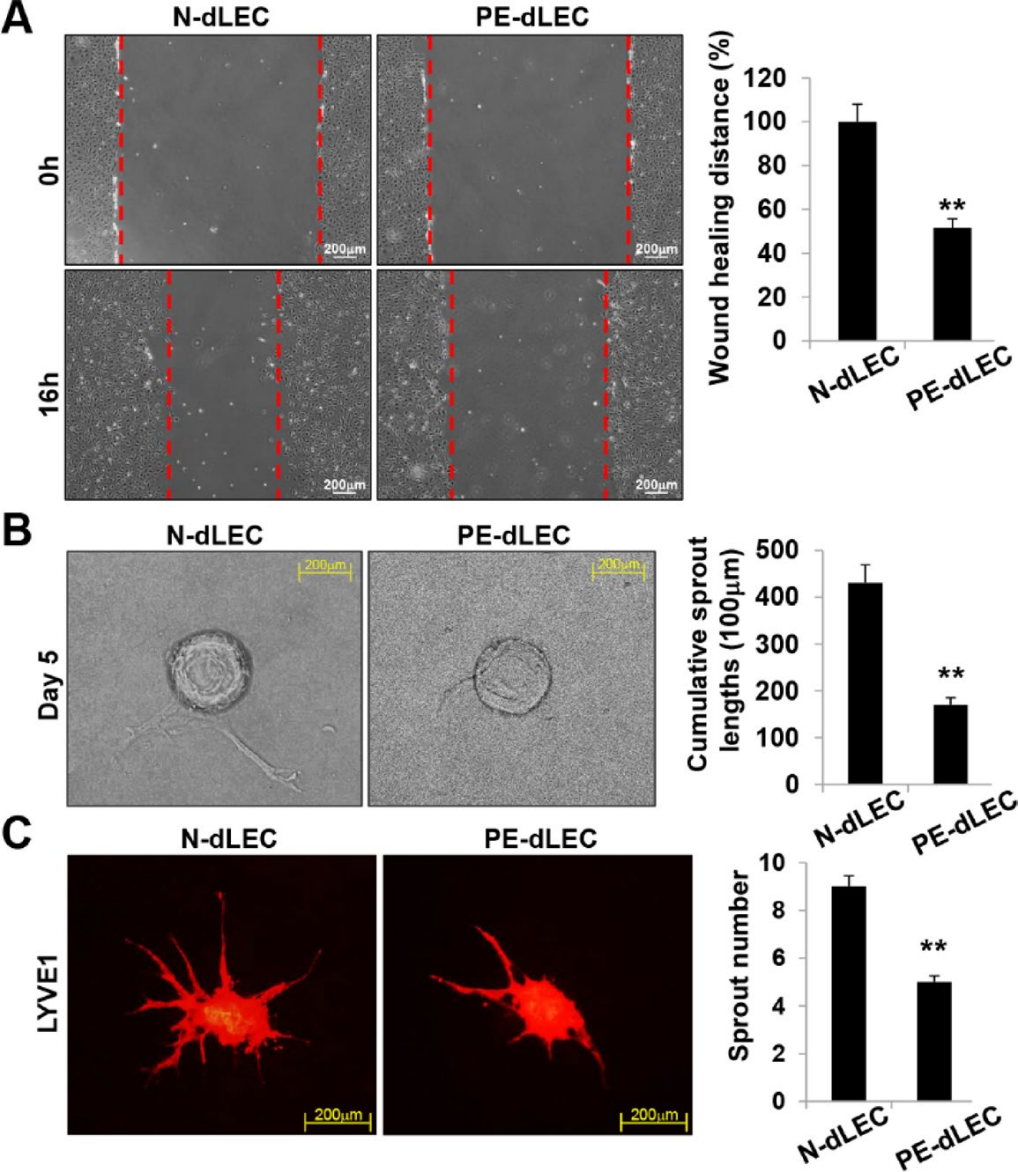
Impaired wound healing, sprouting, and lymphangiogenic activity of dLECs from preeclamptic pregnancies. (**A**) A wound healing assay was performed by scratching confluent dLEC monolayers on 35-mm dishes with a micropipette tip. After removing debris, images were captured at 0 and 16 h after wounding. Wound closure was quantified by measuring the distance between wound edges in five fields per plate using ImageJ. PE-dLECs showed delayed migration into the wounded area, resulting in a larger remaining wound area after 16 h compared with normal controls. (**B**) In vitro 3D lymphangiogenesis assay using fibrin gel-embedded dLEC-coated microbeads. The cumulative sprout length per spheroid was quantified after 5 days, revealing markedly shorter cumulative sprout lengths in PE-dLECs than in N-dLECs. (**C**) dLEC spheroids were embedded in 20% Matrigel-containing fibrin gels and cultured for 48 h. Spheroids were stained for PECAM-1, and the number of sprouts per spheroid was quantified. The number of sprouts formed in PE-dLECs was significantly reduced compared with N-dLECs. All data are presented as the mean ± SEM from three different cell lines (*n* = 7 per group per experiment). Statistical significance was assessed using one-way ANOVA followed by Tukey’s test. **, *p* < 0.01 vs. N-dLEC. dLECs, decidua lymphatic endothelial cells; PE, preeclampsia; PECAM-1, platelet endothelial cell adhesion molecule-1. N-dLECs, dLECs from normal pregnancies; PE-dLECs, dLECs from preeclamptic pregnancies.

Further analysis revealed a marked reduction in wound healing, three-dimensional (3D)-bead sprouting, and 3D-spheroid sprouting activities from the dLEC group compared with controls (Fig. 7A,B). Following wound induction, dLECs from preeclamptic pregnancies showed delayed migration into the wounded area, resulting in a larger remaining wound area after 16 h (Fig. 7A). In the 3D-bead sprouting assay, cumulative sprout length was markedly shorter in PE-dLECs after 5 days of culture (Fig. 7B). Similarly, the number of sprouts formed in the 3D-spheroid assay was reduced in the PE group (Fig. 7C). Collectively, these findings demonstrate that PE-dLECs exhibit impaired lymphangiogenic activity, likely owing to the downregulation of lymphangiogenesis-related genes.

### Reduced CCL21 expression and decreased immune cell chemoattractive activity of PE-dLEC

**Fig. 8:**
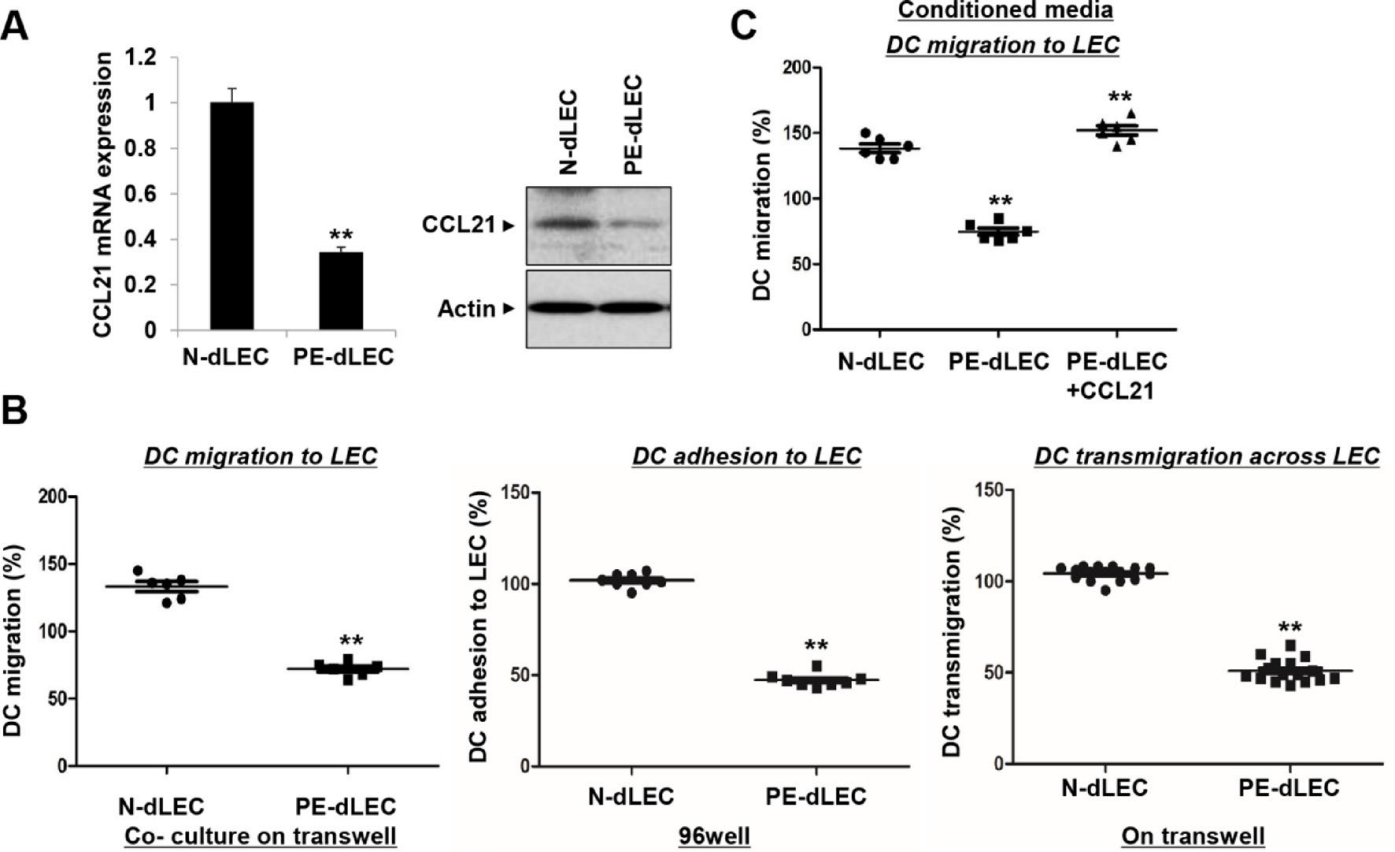
Reduced CCL21 expression and impaired dendritic cell (DC) migration in PE-dLECs. (**A**) PE-dLECs showed significantly reduced expression of CCL21 at both the mRNA and protein levels, as assessed using RT-qPCR and western blot, respectively. (**B**) DC trafficking through LECs was evaluated in three steps: migration, adhesion, and transmigration. PE-dLECs exhibited reduced activity in all three steps. (**C**) Decreased DC migration observed with conditioned media from PE-dLECs was restored by the addition of recombinant CCL21. Data are presented as mean ± SEM. Statistical significance was assessed using one-way ANOVA followed by Tukey’s test. All experiments represent three independent experiments, each performed in duplicate. **, *p* < 0.01 vs. N-dLEC. N-dLECs, dLECs from normal pregnancies; PE-dLECs, dLECs from preeclamptic pregnancies; DC, dendritic cell; dLECs, decidua lymphatic endothelial cells; PE, preeclampsia; CCL 21, chemokine (C-C motif) ligand 21; RT-qPCR, quantitative reverse-transcription PCR.

The expression of CCL21 was decreased in PE-dLECs compared with those from N-dLECs, as confirmed by RT-PCR and western blotting (Fig. 8A). CCL21, expressed by high endothelial venules and LECs, plays a role in directing dendritic cell (DC) trafficking to the cortical T-cell zones of draining lymph nodes^37–39^. To test whether reduced CCL21 in PE-dLECs impairs DC trafficking, we conducted in vitro assays. DC migration, adhesion, and transmigration toward PE-dLECs were all decreased in PE-dLECs compared with N-dLECs (Fig. 8B). This impairment was reversed by the addition of recombinant CCL21, restoring DC mobility to levels observed with N-dLECs (Fig. 8C). These findings indicate that reduced CCL12 expression in PE-dLECs hinders DC trafficking.

### Decreased nitric oxide (NO) production of PE-dLEC

**Fig. 9:**
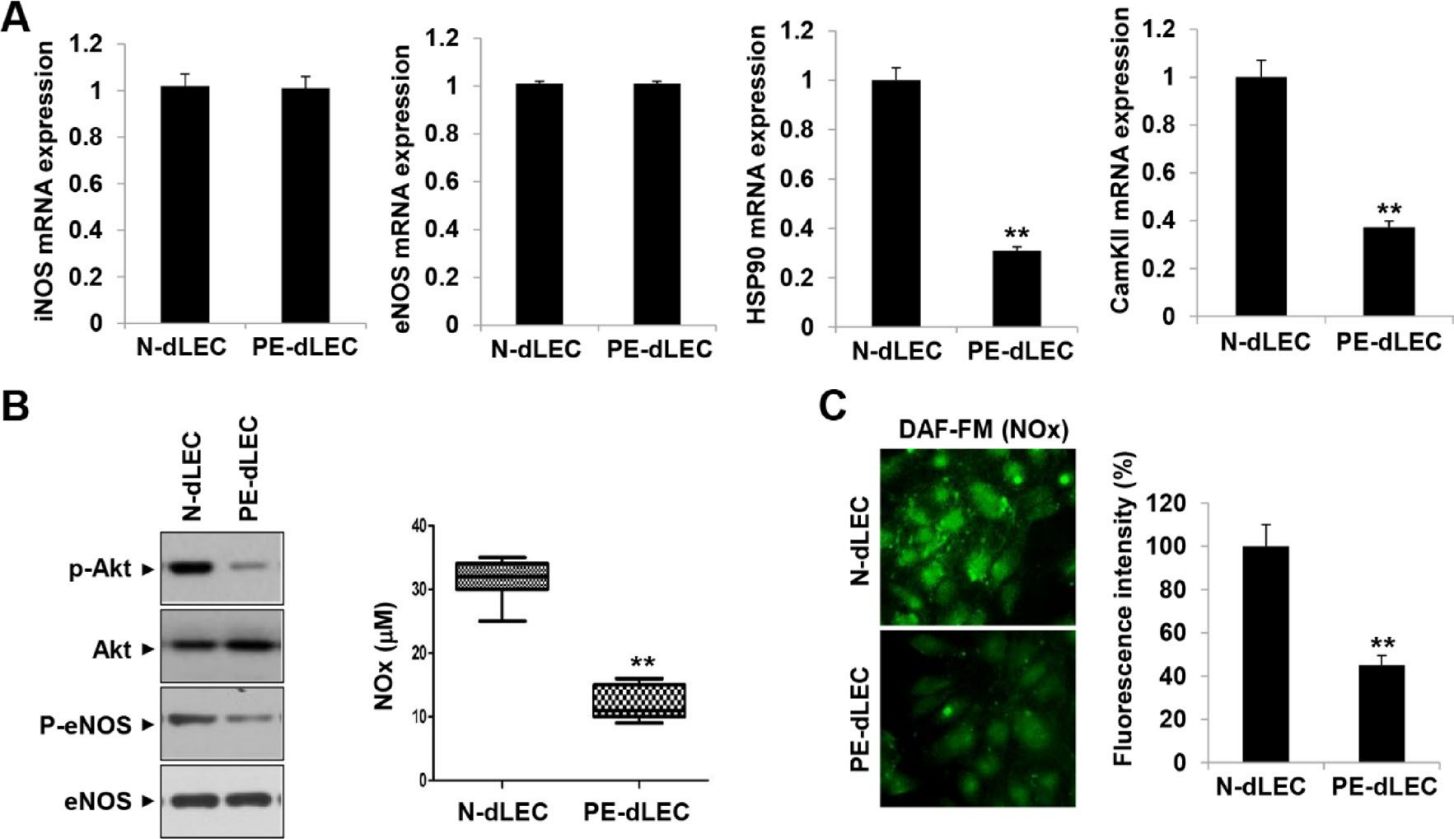
Reduced nitric oxide (NO) production in PE-dLECs. (**A**) RT-qPCR analysis of NO-related genes. mRNA expression levels of iNOS and eNOS showed no differences between PE and normal pregnancies. In contrast, HSP90 and CaMKII expression levels were significantly lower in PE-dLECs compared with normal controls. (**B**, **C**) Representative western blot images of Akt, eNOS, phosphorylated-Akt, and phosphorylated-eNOS in dLECs from normal and PE pregnancies. Quantitative analysis of NO production and fluorescence intensity revealed significantly reduced NO levels in PE-dLECs. All data are presented as the mean ± SEM from three different cell lines. Statistical significance was assessed using one-way ANOVA followed by Tukey’s test. **, *p* < 0.01 vs. N-dLEC. N-dLECs, dLECs from normal pregnancies; PE-dLECs, dLECs from preeclamptic pregnancies; dLECs, decidua lymphatic endothelial cells; PE, preeclampsia; NO, nitric oxide; RT-qPCR, quantitative reverse-transcription PCR; eNOS, endothelial nitric oxide synthase; iNOS, inducible nitric oxide synthase; Akt, Protein Kinase B; CaMKII, Ca²⁺/Calmodulin-Dependent Protein Kinase II.

We analyzed the NO pathway in normal and preeclamptic pregnancies. NO, produced by LECs, plays a key role in regulating vascular permeability and acts as an anti-inflammatory signaling molecule^40,41^. It also regulates T-cell proliferation, as LECs suppress T-cell expansion by increasing NO production in response to proinflammatory cytokines^42^. HSP90 regulates endothelial nitric oxide synthase (eNOS) activity through HSP90-dependent phosphorylation, which is critical for eNOS function^43^. Additionally, CaMKII is a key regulator of NO secretion in endothelial cells^44^. No significant differences were observed in the levels of inducible nitric oxide synthase (iNOS) and eNOS between PE-dLEC and N-dLECs. However, mRNA expression levels of HSP90 and CaMKII were significantly lower in PE-dLECs compared with N-dLECs (Fig. 9A). Both HSP90 and CaMKII interact with and activate downstream signaling molecules Akt and eNOS. Consistently, levels of phosphorylated Akt and eNOS were significantly decreased in PE-dLECs, accompanied by reduced NOx production (Fig. 9B,C). These findings suggest that NO-related gene expression and NOx production are impaired in PE-dLECs, which likely leads to reduced suppression of T-cell activation.

## Discussion

PE is a pregnancy-related hypertensive disorder specific to humans and poses risks to both maternal and fetal health. Although the exact causes of PE remain unclear, emerging data suggest that exaggerated activation of both innate and adaptive immune responses contributes to the breakdown of immune tolerance during pregnancy^13,19,45,46^.

Numerous studies have implicated immune cells, particularly regulatory T cells, macrophages, natural killer cells, and neutrophils, in the pathogenesis of PE^13,47–50^. Lymphatic vessels at the maternal-fetal interface also play crucial roles in regulating immune cell trafficking and antigen presentation, both of which influence the establishment of immune tolerance during pregnancy^22^.

Lymphangiogenesis, the formation of new lymphatic vessels from existing ones, requires LEC proliferation, migration, and tube formation. Liu et al. reported that impaired angiogenesis and lymphangiogenesis in the decidua were associated with PE^51^. Similarly, Rutkowski et al. demonstrated that neutralizing VEGFR-3, a key regulator of lymphangiogenesis, in mice leads to pregnancy failure, resulting in fetal growth restriction and miscarriage^52^. Jung et al. further reported a reduction in the decidual lymphatic system in severe PE and identified an association between granulocyte-macrophage colony-stimulating factor from dLECs and the development of PE^23^.

We conducted the study to demonstrate the lymphangiogenesis activity of dLECs in PE. Compared with N-dLECs, PE-dLECs exhibited reduced tube formation, adhesion, migration, and proliferation. Wound healing and 3D-sprouting capacities were also diminished. Gene expression analyses revealed significant downregulation of pathways involved in lymphatic vessel development, immune cell trafficking, and T-cell activation. In PE-dLECs, DC migration, adhesion, and transmigration were impaired, accompanied by decreased CCL21 expression. However, DC migration was restored upon the addition of CCL21. Furthermore, NO production was reduced in PE-dLECs, despite no changes in eNOS or iNOS expression. This reduction was associated with significantly lower levels of HSP90 and CaMKII, as well as decreased phosphorylation of downstream signaling molecules Akt and eNOS.

Our findings indicate that dysfunctional dLECs in PE are linked to reduced expression of genes including *SOX18*, *NR2F2*, *PROX1*, *LYVE1*, *SEMA3A*, *CCL21*, *HSP90*, and *CAMKII*, which are known to be involved in lymphatic vessel development, immune cell trafficking, and T-cell activation.

The transcription factor SOX18 directly induces PROX1 transcription by binding to its proximal promoter region. Elevated SOX18 expression in blood vascular endothelial cells promotes the expression of PROX1 and other lymphatic endothelial markers. Furthermore, SOX18 is overexpressed in various cancers^53^. In contrast, knockdown of SOX18 in embryos results in a complete inhibition of LEC differentiation from the cardinal vein^54^.

NR2F2, also known as Coup-TFII, functions as a direct activator of PROX1 expression in venous LEC progenitors. It binds to a conserved DNA sequence within the regulatory region of PROX1, a critical step for the initial specification of LECs. Moreover, the sustained expression of PROX1 depends on its interaction with nuclear hormone receptors, likely including NR2F2^55^.

PROX1 is a transcription factor essential for lymphatic system development during embryogenesis and is widely recognized as a marker of lymphatic vessels. Studies using knockout mice models have shown that loss of PROX1 impairs the differentiation of LECs. Beyond development, PROX1 has also been implicated in cancer biology, where it promotes breast cancer invasion and metastasis through interaction with heterogeneous nuclear ribonucleoprotein K and activating the WNT/β-catenin pathway^56^.

LYVE-1 is a type I integral membrane polypeptide that functions as a cell surface receptor for hyaluronan and is primarily expressed on LECs. It plays a pivotal role in facilitating the entry of antigen-presenting DCs and macrophages into initial capillaries by binding to hyaluronan^57^. The LYVE-1-hyaluronan axis appears to regulate this process, as engagement of DCs via LYVE-1 can trigger CCL21 secretion from LECs. In Lyve1−/− mice, this CCL21 release from dermal lymphatic capillaries is disrupted, highlighting LYVE-1’s role in immune cell trafficking^58^.

SEMA3A is a member of the semaphorin family, consisting of secreted or transmembrane proteins that regulate cell movement and adhesion. SEMA3A influences axon guidance, vascular development, immune cell function, and tumor progression through its receptors called neurophilin-1 (Nrp1) and PlexinA1^59–61^. Ricci et al. reported that SEMA3A variants are inherited in families with lymphatic malformations^62^, while Bouvrée et al. demonstrated that SEMA3A-Nrp1-PlexinA1 signaling is required for lymphatic valve formation^63^.

CCL21 is stored in the intracellular compartments of resting LECs and is continuously secreted under normal conditions. LEC-derived CCL21 facilitates DC transmigration by binding to the CCR7 receptor^37,64–66^. For mouse dermal DCs, mobilization is regulated by the CCL21–CCR7 axis, which guides DC chemotaxis toward lymphatics, promotes transmigration through the lymphatic endothelium, and enables intralymphatic crawling in response to lymph flow. This highlights the importance of directional migration and adhesion in this process^67^. In mice lacking CCL21 expression, leukocyte localization is impaired, causing T cell–DC interactions to occur outside the usual confines of the T cell zone^68^.

HSP90 is a chaperone protein that facilitates proper protein folding, stabilizes proteins under heat stress conditions, and aids in protein degradation. In endothelial cells, HSP 90 also regulates the balance between NO and superoxide anion production by modulating eNOS activity. Inhibition of HSP90-dependent signaling in eNOS shifts its enzymatic activity from NO production to superoxide anion generation, leading to increased oxidative stress^69,70^. Prangsaengtong et al. demonstrate that the interaction between eNOS and HSP90 is crucial in modulating eNOS levels and regulating lymphangiogenesis in human LECs in vitro^71,72^. HSP90 has been extensively studied as a therapeutic target in a range of human diseases, including neurodegenerative diseases, pulmonary/respiratory diseases, and various cancers^73^. HSP90 expression is linked to T cell activation and has potential as a therapeutic target in chronic inflammatory diseases such as rheumatoid arthritis^74^.

CaMKII is a serine/threonine-specific protein kinase modulated by the Ca^2+^/calmodulin complex. CaMKII plays a role in maintaining calcium homeostasis, which contributes to controls NO synthesis^75,76^. NO regulates the adaptive immune response by influencing the differentiation and activation of T cells, including the fine-tuning of cytotoxic T cell generation^77–83^. Elevated levels of CaMKII have been reported in various malignant diseases^84,85^.

In summary, these findings indicate that impaired lymphangiogenesis in PE may result from the downregulation of genes essential for LEC differentiation, vessel maturation, and immune regulation. Reduced expression of SOX18, NR2F2, and PROX1 likely disrupts the transcriptional network required for lymphatic vessel development, while decreased SEMA3A expression may impair lymphatic patterning and valve formation. Lower levels of LYVE-1 and CCL21 may further hinder immune cell trafficking and the clearance of inflammatory mediators. In addition, suppression of HSP90 and CaMKII could weaken the Akt–eNOS signaling pathway, leading to endothelial dysfunction and reduced nitric oxide bioavailability. Together, these molecular alterations may attenuate lymphangiogenic capacity and promote a proinflammatory decidual environment, thereby contributing to the pathophysiology of PE. Further research is required to elucidate how changes in lymphangiogenesis-related genes modulate dLEC function and affect pregnancy outcomes.

Apart from structural impairment of lymphangiogenesis, these molecular alterations may have broader implications for immune regulation within the decidua. LECs are increasingly recognized as active modulators of immune tolerance through their interactions with DCs and Treg cells. Tregs are essential for maintaining maternal–fetal immune tolerance, as they suppress excessive activation of both innate and adaptive immunity during early gestation^86^. Under physiological conditions, LEC-derived factors such as CCL21 and NO facilitate the recruitment and maintenance of Tregs within lymphatic-rich regions, thereby supporting local immune tolerance. In PE, the reduced expression of CCL21, HSP90, and CaMKII may impair these mechanisms, leading to diminished Treg migration and function and promoting a shift toward a proinflammatory state. This dysregulated LEC–Treg crosstalk could therefore represent a key link between defective lymphangiogenesis and the heightened inflammatory environment characteristic of PE.

The genetic differences in LECs at the maternal-fetal interface have not been studied. With increasing recognition of lymphatic vessels in the decidua^16,22,25^ and the ability to isolate primary human dLECs from both normal and pre-eclamptic pregnancies, it is now possible to study their genetic and functional characteristics under both normal and pathological conditions. Previous studies have identified differences in angiogenesis, lymphangiogenesis, and lymphocyte differentiation without investigating gene expression levels between normal and pre-eclamptic pregnancies^16,23,25,51^. To our knowledge, this is the first study to demonstrate the impaired functions and genetic differences of dLECs in PE. Our findings enhance current understanding of the role of LECs at the maternal-fetal interface in the pathogenesis of PE.

**Fig. 10:**
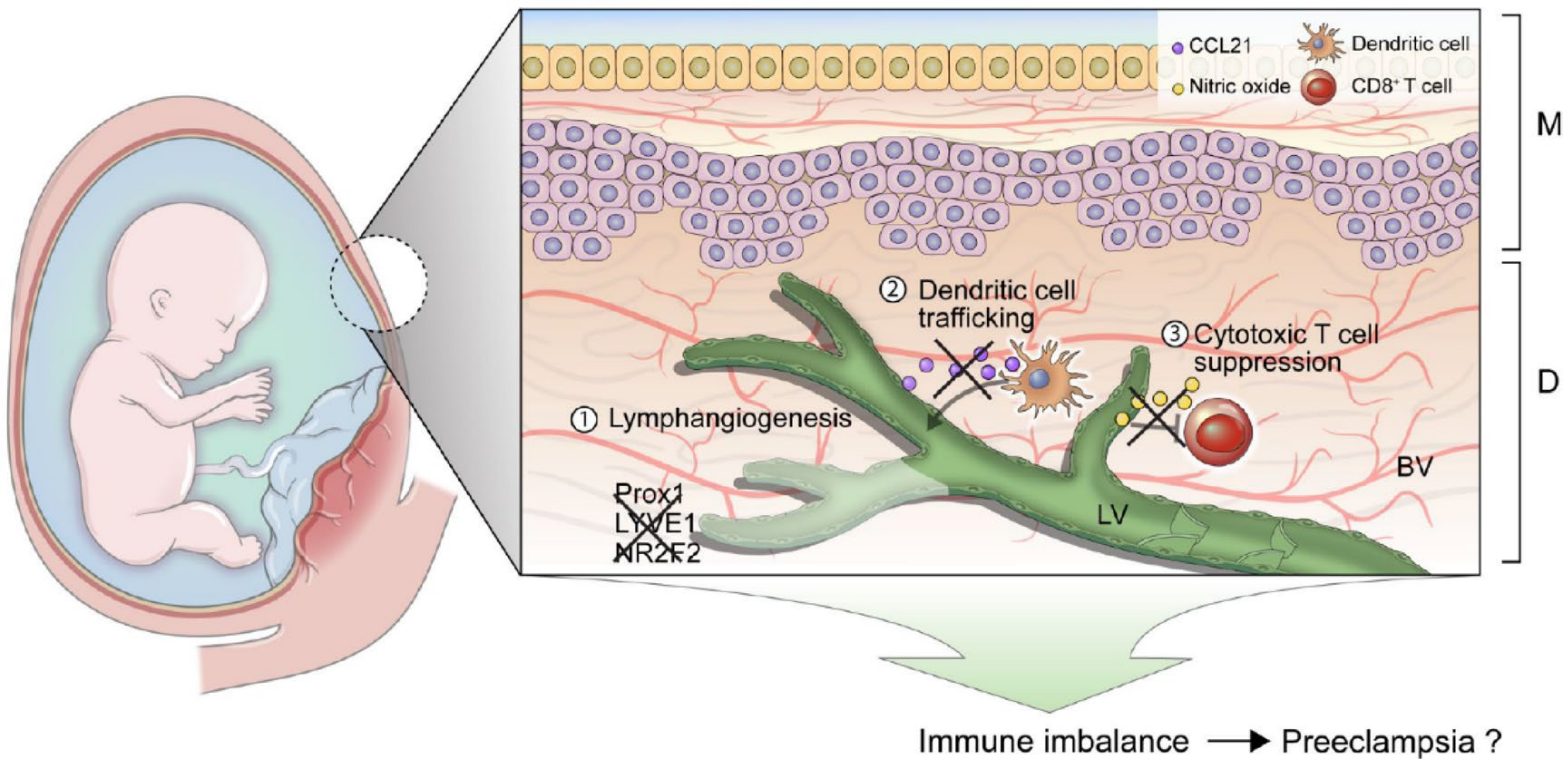
Proposed model of immune and lymphatic dysfunction at the maternal-fetal interface in preeclampsia. This schematic illustrates the functional and genetic differences observed in PE-dLECs. In PE-dLECs, reduced expression of lymphangiogenesis-related genes leads to impaired lymphatic vessel formation and functional deficits. Decreased CCL21 expression disrupts DC trafficking, while reduced NO levels impair the suppression of cytotoxic T cells. Finally, these changes may contribute to immune imbalance and the pathogenesis of preeclampsia. LV, lymphatic vessel; BV, blood vessel; M, amnion chorion; D, decidua; dLECs, decidual lymphatic endothelial cells; PE, pre-eclampsia; NO, nitric oxide; CCL 21, chemokine (C-C motif) ligand 21; DC, dendritic cell.

In conclusion, our findings highlight immune imbalance as a key contributor to the etiology of PE, supported by three major observations. First, reduced expression of lymphangiogenesis-related genes in PE-dLECs impairs lymphatic vessel formation, leading to decreased migration, adhesion, and tube formation capacity. Second, downregulation of CCL21 disrupts DC trafficking. Impaired DC migration may lead to insufficient antigen presentation to T cells in the lymph nodes, which hinders the initiation of adaptive immune responses and may contribute to dysregulated immune tolerance. Third, reduced NO production diminishes the suppression of cytotoxic T cell activity, promoting immune overactivation (Fig. 10).

While there is a growing interest in developing new pharmacological treatments for PE, therapeutic options remain limited, and the use of novel therapies may pose safety risks for the developing fetus^87^. Therefore, further studies are needed to investigate the stimuli and downstream mechanisms that alter dLECs in PE and develop targeted drug delivery methods that do not adversely affect fetal development.

## Methods

### Study population and sample collection

Deliveries between 33 and 38 weeks of gestation at Severance Hospital from January 2019 to February 2021 were included in this study. Pregnant participants were dichotomized into the PE and control groups (*n* = 15 per group) (Table 1). Fetal chorioamniotic membranes containing decidual tissue were collected at the time of cesarean delivery, following fetal expulsion, from both pre-eclamptic and normal pregnancies. Severe PE was diagnosed according to the criteria of the American College of Obstetricians and Gynecologists^88^. Gestational age-matched controls were selected from individuals who delivered for other indications and had no history of PE or chronic hypertension. The exclusion criteria included a clinical or pathological diagnosis of chorioamnionitis, maternal autoimmune disease, or multiple pregnancies. This study was approved by the Institutional Review Board of Severance Hospital (approval no. 4-2016-0450), and written informed consent was obtained from all participants. All the methods were carried out in accordance with relevant guidelines and regulations.

**Table 1:** Patient characteristics.

	Normal pregnancy (*n* = 15)	Preeclampsia (*n* = 15)	*p* value
Maternal age (years)	32.5 ± 5.1	33.4 ± 4.7	0.578
Gestational age at delivery (weeks)	38.4 ± 1.5	35.9 ± 2.2	< 0.001
Gravida	1.7 ± 0.5	2.1 ± 1.2	0.520
Blood pressure (mmHg)			
Systolic blood pressure	109.6 ± 11.3	145.4 ± 13.6	< 0.001
Diastolic blood pressure	72.1 ± 6.5	100.6 ± 11.3	< 0.001
Pre-pregnancy BMI (kg/m^2^)	23.1 ± 5.2	23.8 ± 4.1	0.375
Birthweight (g)	3208 ± 426.0	2223.8 ± 632.8	< 0.001
Small gestational age newborn (n)	1	9	0.018

BMI, body mass index.

### Isolation and cultivation of primary human dLECs and DCs

Decidual tissues were aseptically scraped from fetal chorioamniotic membranes using sterile forceps and scissors, minced into 1–2 mm pieces, and digested with 0.25% collagenase II at 37 °C for 2 h. The digested tissues were filtered through a 100 µM cell strainer once, and the resulting cell suspension was cultured on fibronectin-coated culture dishes (Sigma-Aldrich, St. Louis, MO, USA) in endothelial basal medium (EBM; Lonza, Walkersville, MD, USA) supplemented with 10% FBS and other factors, as previously described^89^.

After 48 h in culture, unbounded cells were removed by washing with PBS, and the remaining cells were collected for flow cytometry. Cells were stained with antibodies against LYVE1 (Abcam, Cambridge, England) and CD31 (DAKO, Glostrup, Denmark), and sorted using a FACSAria flow cytometer (BD Biosciences, Bedford, MA, USA) with FACSDiva software (BD Biosciences, Bedford, MA, USA). To confirm the identity of isolated cells as dLECs, we performed immunostaining with the lymphatic markers LYVE-1 (Abcam, Cambridge, England) and Prox-1 (Reliatech GmbH, Wolfenbüttel, Germany) and the pan-endothelial marker CD31 (DAKO, Glostrup, Denmark). Cells demonstrating triple-positive fluorescence were identified as dLECs.

Peripheral blood mononuclear cells were obtained from leukocyte filters connected to blood donation units from healthy donors who had provided informed consent^90^. CD14^+^ monocytes were isolated using CD14 MicroBeads and AutoMACS equipment (Miltenyi Biotec, Madrid, Spain), following the manufacturer’s instructions. Human IL-4-DCs were generated as described previously^91^.

### Flow cytometry

dLECs were gently detached from culture plates using PBS containing 2 mM EDTA, rinsed two to three times with PBS, and resuspended in PBS containing 3% BSA. The cells were then incubated for 30 min on ice with fluorochrome-tagged monoclonal antibodies (BD Biosciences, Bedford, MA, USA) against surface LYVE1 (Abcam, Cambridge, England), CD31 (DAKO, Glostrup, Denmark). Labelled cells were sorted using a FACSAria flow cytometer equipped with FACSDiva software (BD Biosciences, Bedford, MA, USA).

### RNA-sequencing for gene expression analysis

Total RNA was isolated using TRIzol reagent (Invitrogen, Carlsbad, CA, USA), and RNA quality was assessed using an Agilent 2100 Bioanalyzer with the RNA 6000 Nano Chip (Agilent Technologies, Amstelveen, The Netherlands). RNA quantification was performed using the ND2000 Spectrophotometer (Thermo Fisher Scientific Inc., Wyman Street, Waltham, MA, USA).

For dLECs derived from the pre-eclamptic (*n* = 3) and normal (*n* = 3) pregnancies, library construction was performed using the QuantSeq 30-mRNA-Seq Library Prep Kit (Lexogen, Inc., Wien, Austria), according to the manufacturer’s instructions. Briefly, 500 ng of total RNA was hybridized with an oligo-dT primer containing an Illumina-compatible sequence at the 5′ end and subjected to reverse transcription. After degradation of the RNA template, second-strand synthesis was initiated using a random primer with an Illumina-compatible linker sequence at the 5′ end. The resulting double-stranded library was purified using magnetic beads to remove residual components. The library was then amplified to incorporate full adapter sequences required for cluster generation and subsequently purified from PCR components. High-throughput sequencing was performed on the NextSeq 500 (Illumina, Inc., San Diego, CA, USA) using single-end 75 bp reads. QuantSeq 30-mRNA-Seq reads were aligned using Bowtie2^92^. The Bowtie2 indices were generated from either the genome assembly or representative transcript sequences for alignment. The resulting alignment files were used for transcript assembly, abundance estimation, and differential gene expression analysis. Differentially expressed genes were identified based on read counts (RCs) from both unique and multiple alignments, using coverage data generated with BEDtools^93^. RC data were normalized using the quantile normalization method implemented in the EdgeR within the R statistical environment^94^, as part of the Bioconductor framework^95^. We showed that *SOX18*, *NR2F2*, *PROX1*, *LYVE1*, *SEMA3A*, *CCL21*,* HSP90*, and *CAMKII* expression is downregulated in PE-dLEC (BioProject ID: PRJNA1345170). Gene classification and functional annotation were performed using DAVID (http://david.abcc.ncifcrf.gov/, accessed on 21 July 2018) and the Medline database (http://www.ncbi.nlm.nih.gov/, accessed on 21 July 2018).

### Amelogenin X (*AMELX*), *AMELY*, and *SRY* gene analysis

Genomic DNA from human dLECs was isolated using the QIAGEN Genomic DNA purification kit (Qiagen, Hilden, Germany), following the manufacturer’s instructions. qPCR assays were performed to detect the *AMELX*, *AMELY*, and *SRY* genes in the extracted DNA^96,97^. PCR amplification was carried out using a Veriti Thermal Cycler (Applied Biosystems; ABI) under the following conditions: initial denaturation at 95 °C for 2 min, followed by 40 cycles of 95 °C for 1 min, 54 °C for 2 min, and 74 °C for 1 min. PCR products were separated on an agarose gel stained with ethidium bromide and visualized using a gel documentation system (GDS). The GDS included a UV transilluminator connected to a computer for visualization, analysis, and data storage. Primers used for gene amplification are listed in Table 2.

**Table 2:** Primers used for gene amplification.

AMEL	Sense- acctcatcctgggcaccctgg	LYVE1	Sense- atggaaactagcaccatgtc
	Antisense- aggcttgaggccaaccatcag		Antisense- gaagaggagagcaagcacta
SRY	Sense- tccaggaggcacagaaatta	CCL21	Sense- agtctggcaagaagggaaag
	Antisense- tcttgagtgtgtggctttcg		Antisense- gggtctgtggctgttcagt
SEMA3A	Sense- tggaagtcattgacacagag	iNOS	Sense-gctgaaattgaatgaggagc
	Antisense- aagtctctgtaccagacctt		Antisense -cttcgcctcgtaaggaaata
NR2F2	Sense- agctgcctcaaggccatagt	eNOS	Sense- atgttaccatggcaaccaa
	Antisense- ggttggggtactggctccta		Antisense- gaaaatgtcttcgtggtagc
SOX18	Sense- ccagtacctcaactgcagcc	HSP90	Sense- ggcaaggacatctctacaaa
	Antisense- ctctcctctgggcaggacat		Antisense- ccttaattcgtcgaagcatg
PROX1	Sense-aaggcaacaacaaagaaaga	CaMKII	Sense-tcaacaatggggactttgaa
	Antisense-agactttgaccacagtgtcc		Antisense- gaaaatccatcccttccact
GAPDH	Sense- atggggaaggtgaaggtcg		
	Antisense-ggggtcattgatggcaacaata		

AMEL, Amelogenin; LYVE1, Lymphatic Vessel Endothelial Hyaluronan Receptor 1; SRY, Sex Determining Region Y; CCL21, C-C Motif Chemokine Ligand 21; SEMA3A, Semaphorin 3 A; iNOS, Inducible Nitric Oxide Synthase; NR2F2, Nuclear Receptor Subfamily 2 Group F Member 2; eNOS, Endothelial Nitric Oxide Synthase; SOX18, SRY-Box Transcription Factor 18; HSP90, Heat Shock Protein 90; PROX1, Prospero Homeobox 1; CaMKII, Calcium/Calmodulin-Dependent Protein Kinase II; GAPDH, Glyceraldehyde-3-Phosphate Dehydrogenase.

### RT-qPCR

Total RNA was isolated from dLECs using TRIzol reagent (Invitrogen). mRNA expression of human genes was quantified using the Power SYBR Green RNA-to-CT™ 1-Step kit (Applied Biosystems, Foster City, CA, USA) and StepOnePlus™ (Applied Biosystems), according to the manufacturers’ instructions. The cycling conditions were as follows: reverse transcription at 48 °C for 30 min, initial denaturation at 95 °C for 10 min, followed by 40 cycles of 95 °C for 15 s and 55 °C for 1 min. Gene expression levels were determined using cycle threshold (Ct) values. The difference between the Ct values of the target gene and the reference gene (*GAPDH*) was calculated, and relative expression was expressed as the ratio of RNA in each group compared with that of the calibrated sample. Primers used for gene amplification are listed in Table 2.

### In vitro tube formation assay

Tube formation was assayed as previously described^89^. Briefly, 250 µL of Matrigel (BD Biosciences, Bedford, MA, USA) was added to 16-mm diameter tissue culture wells and allowed to polymerize for 30 min at 37 °C. The dLECs were harvested by trypsinization, resuspended in EBM, and seeded and plated onto the Matrigel at a density of 1.2 × 10^5^ cells/well. Matrigel cultures were incubated at 37 °C and photographed at various time points using ×200 magnification. The area covered by the tube network was quantified using an optical imaging approach: images were scanned into Adobe Photoshop and analyzed with ImageJ software (National Institutes of Health).

### Cell-matrix adhesion assay

Cell-matrix adhesion assays were performed as previously described^98^. The 96-well plates were coated overnight at 4 °C with 0.1 mg/mL human fibronectin (Sigma-Aldrich, St Louis, MO, USA). dLECs in adhesion buffer (serum-free media) were seeded at 10^5^ cells/well in a 100 µL volume and incubated for 30 min at 37 °C. Non-adherent cells were removed after two gentle washes with PBS. Adherent cells were fixed, stained with H&E, and quantified in triplicate by counting cells in five randomly selected fields per well using an Axiovert 100 microscope (Carl Zeiss Micro-Imaging, Thornwood, NY, USA). Results are representative of three independent experiments, each performed in duplicate.

### Cell migration assay

Cell migration was assayed using a Transwell system (Corning Costar, Acton, MA) with 6.5-mm diameter polycarbonate filters (8 mm pore size). The lower surface of each filter was coated with 10 mg/mL fibronectin (Sigma-Aldrich, St Louis, MO), and 3% (w/v) BSA was used as a control for non-specific binding. dLECs (1 × 10^5^) were seeded onto chemotaxis filters in EBM plus 0.5% FBS. Recombinant human VEGF-C (Upstate Biotechnology, Lake Placid, NY) was then added to the lower chamber. After the 5-h migration period, non-migrating cells were completely removed from the top surface of the membrane. Migrating cells adhering to the undersurface of the filters were measured by H&E staining and quantified using Kodak 1D software (Eastman Kodak, Rochester, NY). Results are representative of three independent experiments performed in duplicate.

### Cell proliferation assay

dLECs (1 × 10^3^ cells) were seeded into 96-well plates and cultured for 72 h. At designated time points (24 h, 48 h, and 72 h), cell viability was assessed using the 3-(4,5-dimethylthiazol-2-yl)-2,5-diphenyl tetrazolium bromide (MTT) assay. For each time point, 20 µL of MTT reagent (5 mg/mL) was added to each well and incubated for 4 h. Following incubation, the supernatant was removed, and the cells were treated with 150 µL of DMSO. Absorbance (optical density, OD) was measured at 490 nm using a microplate reader. The experiments were performed in triplicate with three different cell lines.

### Wound healing assay

The wound healing assay was performed by scratching confluent dLECs on 35-mm dishes with micropipette tips, and the cells were washed to remove debris. Images were captured at 0 and 16 h after wounding. For quantitative analysis, five fields per plate were photographed, and distances between the front lines were measured using ImageJ software (National Institutes of Health). Each assay was repeated three times.

### 3D bead sprouting assay

Cytodex 3 microcarrier beads (GE Healthcare Bio-Sciences Corp, Piscataway, NJ) were coated with dLECs (mixed at a ratio of 400 cells/bead) in Microvascular Endothelial Cell Growth Medium-2 MV (Lonza). The coated beads were embedded in 2 mg/mL fibrin gels in 48-well plates by mixing 2 mg/mL fibrinogen (Chemicon Inc., USA) in HBSS with 1 U/mL thrombin and 150 mg/mL aprotinin (Sigma-Aldrich, St Louis, MO). Endothelial Cell Growth Medium-2 (Lonza) containing WI-38 cells (11,000 per well) was added to each well. Cultures were maintained for 5 days with medium changes every other day, followed by fixation with 4% paraformaldehyde for 1 h at room temperature (approximately 22 °C). Bright-field images were captured using an Axiovert 200 microscope (Zeiss) at 5× magnification, and sprout lengths were quantified using ImageJ software. The experiments were performed in triplicate with three different cell lines.

### 3D spheroid sprouting assay

Three thousand dLECs were cultured in round-bottom 96-well plates (Nunc, Rochester, USA), precoated with 0.8% agarose for 1 day for spheroid formation. The spheroids were then collected and embedded in 20% Matrigel-containing (BD Biosciences, Bedford, MA) fibrin gels (3 mg/mL fibrinogen, 2 U/mL thrombin, and 200 mg/mL aprotinin). The spheroids were cultured in LEC medium for 48 h. Spheroids were then fixed in 4% PFA for 1 h at room temperature (approximately 22 °C) and identified using staining with mouse anti-CD31 monoclonal antibody (clone: JC70A, 1:100 dilution, DAKO, Denmark). The experiments were performed in triplicate with three different cell lines.

### DC migration to dLECs

Cell migration was assayed using the Transwell system (Corning Costar, Acton, MA) with 6.5 mm diameter polycarbonate filters (8 mm pore size). PE-dLECs or N-dLECs (3 × 10^4^) were seeded into the lower chamber and incubated for 24 h. The lower surface of the upper chamber was coated with 10 mg/mL fibronectin (Sigma-Aldrich, St Louis, MO), and human DCs (1 × 10^5^) were seeded into the upper chamber in EBM supplemented with 0.5% FBS. After a 12-h migration period, non-migrating cells were removed from the upper membrane surface. Migrated cells adhering to the underside of the filters were stained with H&E and quantified using Kodak 1D software (Eastman Kodak, Rochester, NY, USA). Results are representative of three different experiments performed in duplicate.

### DC to dLEC adhesion assays

dLECs were plated onto 2% fibronectin-coated 96-well plates at a density of 1 × 10^4^ cells/well. Human DCs were then added (5 × 10^4^ cells/mL, 200 µL/well) to the confluent dLEC monolayers and incubated for 30 min. Thereafter, wells were washed three times with PBS, fixed, and stained with DiffQuick (Baxter Healthcare Corp., McGraw Park, IL, USA). Adherent cells were quantified by counting five randomly selected optical fields per well. Each condition was tested in duplicate and represents three independent experiments.

### DC transmigration across dLECs

Cell transmigration was assayed using the Transwell system (Corning Costar, Acton, MA) with 6.5 mm diameter polycarbonate filters (8 mm pore size). The lower surface of the upper chamber was coated with 10 mg/mL fibronectin (Sigma-Aldrich, St Louis, MO). PE-dLECs or N-dLECs (1 × 10^4^) were seeded onto chemotaxis filters in EBM with 20% FBS. After 24 h, human DCs (1 × 10^5^) were added to the confluent dLEC monolayer and incubated for 12 h. Non-migrating cells were removed from the upper membrane surface, and migrated cells on the undersurface were fixed, stained with H&E, and quantified using Kodak 1D software (Eastman Kodak, Rochester, NY). Results are representative of three independent experiments performed in duplicate.

### DC migration to dLEC using conditioned media

Cell migration was assayed using the Transwell system (Corning Costar, Acton, MA) with 6.5 mm diameter polycarbonate filters (8 mm pore size). The lower surface of the upper chamber was coated with 10 mg/mL fibronectin (Sigma-Aldrich, St Louis, MO, USA), and human DCs (1 × 10^5^) were seeded onto the chemotaxis filters in EBM with 0.5% FBS. Conditioned media from PE or normal pregnancies collected after 24 h of culture in EBM with 1% FBS were added to the lower chamber, with or without recombinant human CCL21 (250 ng/mL; R&D Systems, Minneapolis, MN, USA). After a 12-h migration period, non-migrating cells were removed from the upper surface of the membrane. Migrated cells on the undersurface were fixed, stained with H&E staining, and quantified using Kodak 1D software (Eastman Kodak, Rochester, NY, USA). Results represent three independent experiments performed in duplicate.

### Nitrate/nitrite determination

At the end of incubation, culture supernatants were collected by centrifugation at 10,000 ´ g for 10 min using a Centra MP4R centrifuge (International Equipment, Needham Heights, MA, USA). Nitrate/nitrite levels were measured using a modified Griess reaction^99^. Nitrate was first reduced to nitrite using nitrate reductase, followed by a 45-min incubation. Samples were then mixed with a solution consisting of equal volumes of 14 mM dapsone (4, 4’-diamino-diphenylsulfone) and 4 mM N-(1-naphthyl) ethylenediamine. After a 5-min color development, absorbance was measured at 550 nm using a Bio-Rad plate reader (Hercules, CA). Nitrate/nitrite levels were also assessed using a chemiluminescence assay^99^, where nitrate was enzymatically reduced to nitrite and quantified by chemiluminescence. Nitrite concentration was determined as the difference in signal with and without 0.5% sulfanilamide in 0.1 M HCl.

### NO measurements with 4-amino-5-methylamino-2′,7′-difluorofluorescein diacetate (DAF-FM) diacetate

Fluorescence measurements were performed following published protocols^99^. Cells cultured on a chambered borosilicate cover-glass system were incubated in serum-free and phenol red-free EBM medium for 60 min. The cells were then loaded with 5 mM DAF-FM diacetate (Invitrogen, Carlsbad, CA) for 30 min at 37 °C. After loading, the cells were washed with fresh medium and treated with agmatine for 10 min, with or without rauwolscine, at room temperature (approximately 22 °C). Fluorescence was measured using a Leica DMIRBE (Exton, PA, USA) inverted epifluorescence/Nomarski microscope equipped with Leica TCS-NT/SP1 laser confocal optics and appropriate filters.

### Western blotting

Cell lysates were fractionated by sodium dodecyl sulphate–polyacrylamide gel electrophoresis and transferred to polyvinylidene fluoride membranes. After blocking, the membranes were incubated with primary antibodies against CCL21 (chemokine ligand 21), P-AKT, AKT, P-eNOS, eNOS, and ACTIN. Immunoreactive bands were visualized using a chemiluminescent reagent as recommended by Amersham Biosciences, Inc. (GE Healthcare, Chicago, IL, USA).

### Statistical analyses

All experiments were repeated at least three times. Data are presented as means and standard error, and statistical comparisons between groups were performed using one-way ANOVA followed by Tukey’s test.

## Supplementary Information

Below is the link to the electronic supplementary material.

Supplementary Material 1

## Data Availability

The datasets generated and/or analysed during the current study are available in the NCBI repository, [BioProject ID: PRJNA1345170]
