# Supplementary material for: Immune regulation and lymphangiogenesis by lymphatic endothelial cells in the decidua in severe preeclampsia
Source: Sci Rep. 2026 Jan 13;16:5181. doi: 10.1038/s41598-026-35667-3 (PMC12881592; doi:10.1038/s41598-026-35667-3)
Supplement: Supplementary file 1 — Supplementary Material 1 [file 41598_2026_35667_MOESM1_ESM.pdf]

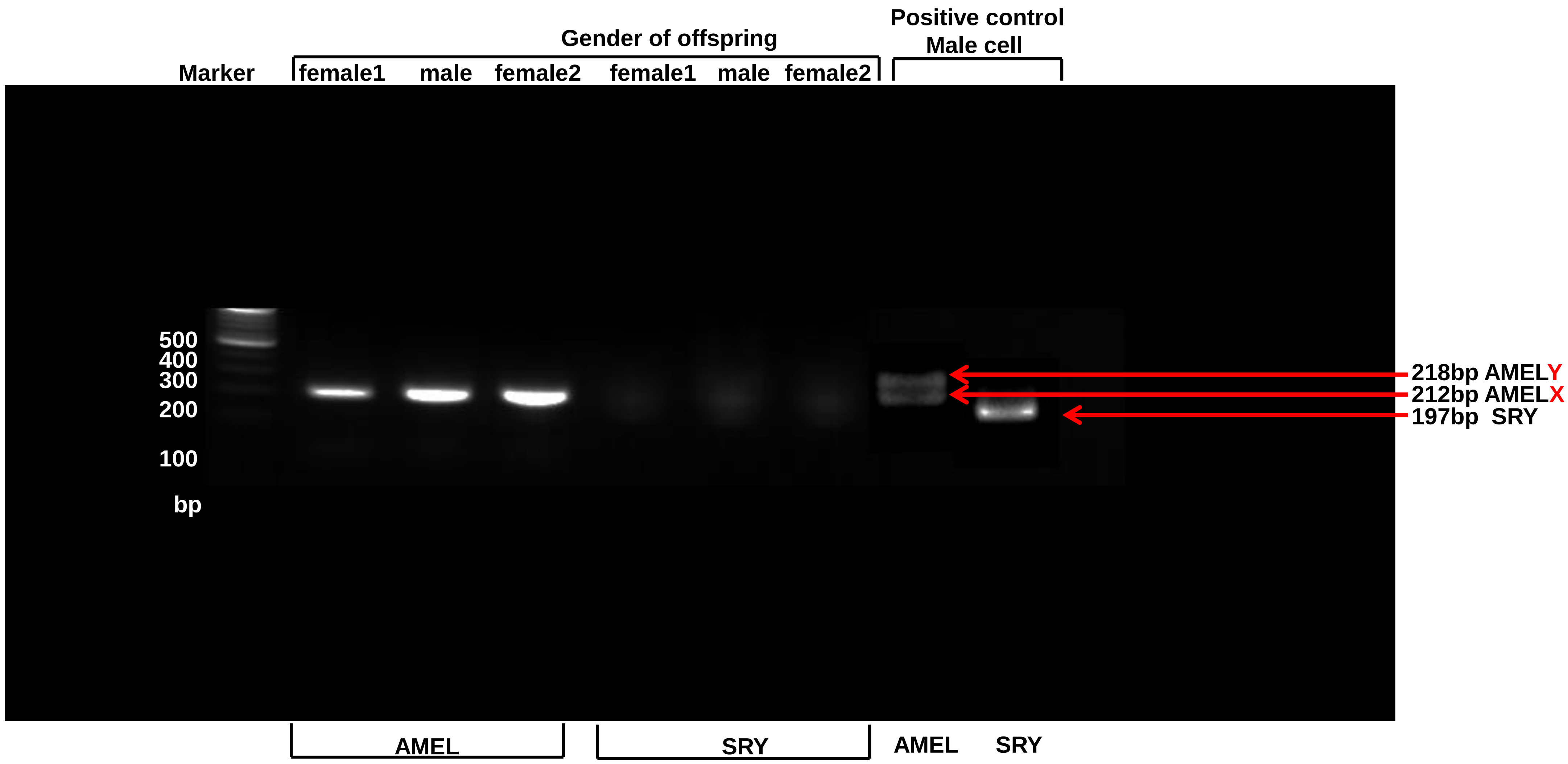

Supplementary figure 1. Confirmation of the maternal origin of isolated dLECs using qPCR.
PCR amplification of the AMEL and SRY genes was performed on genomic DNA extracted from isolated dLECs. Human skin lymphatic endothelial cells from a male donor were used as a positive control. The control sample showed two AMEL bands (218 bp for AMELX and 212 bp for AMELY) and a single SRY band at 197 bp. In contrast, all three dLEC samples (two from pregnancies with female fetuses and one with a male fetus) exhibited only the AMELX band (212 bp) without SRY amplification, confirming that the isolated cells were of maternal origin. dLECs, decidual lymphatic endothelial cells; AMEL, Amelogenin; SRY, sex-determining region Y.

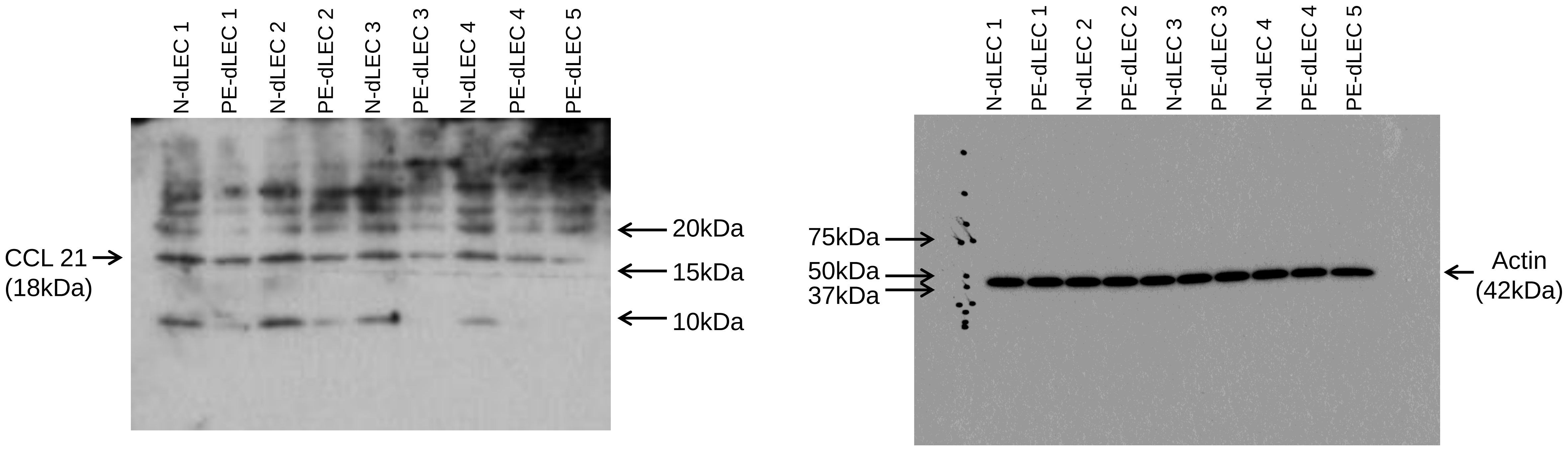

Supplementary figure 2. Reduced CCL21 expression in PE-dLECs. PE-dLECs showed significantly reduced expression of CCL21 protein levels. Cell lysates were subjected to western blotting by using IgGs against CCL21. The membranes were then stripped and reprobed with IgGs against actin to estimate the total protein loaded. Four normal samples and five PE samples were analyzed.

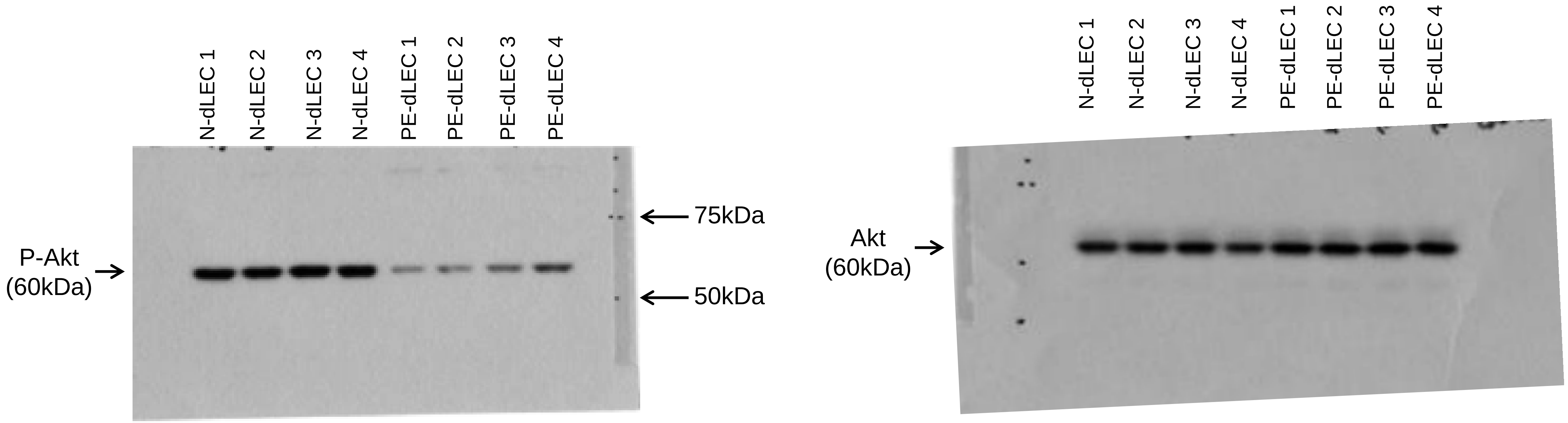

Supplementary figure 3. Reduced Akt phosphorylation in PE-dLECs. PE-dLECs showed significantly reduced phosphorylation of Akt. Cell lysates were subjected to western blotting by using IgGs against pAKT. The membranes were then stripped and reprobed with IgGs against AKT to estimate the total protein loaded. Four normal and PE samples were analyzed.

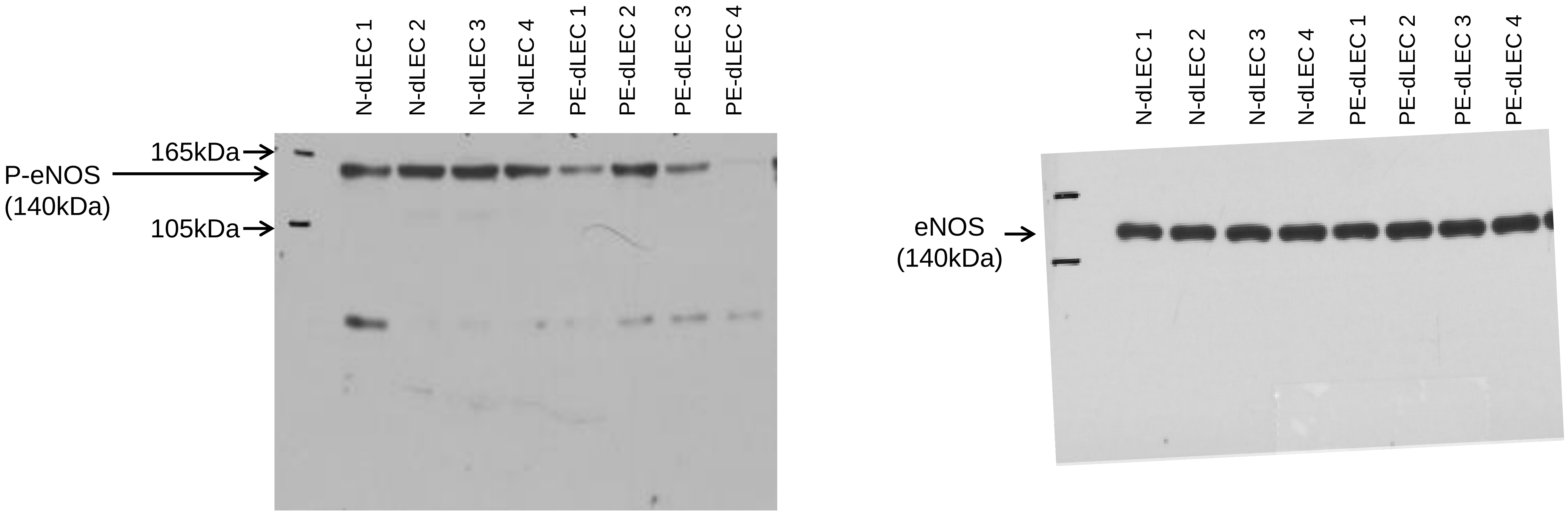

Supplementary figure 4. Reduced eNOS phosphorylation in PE-dLECs. PE-dLECs showed significantly reduced phosphorylation of eNOS. Cell lysates were subjected to western blotting by using IgGs against p-eNOS. The membranes were then stripped and reprobed with IgGs against eNOS to estimate the total protein loaded. Four normal and PE samples were analyzed.
